# Soil Inorganic Electron Acceptors and Carbon Substrates Hierarchically Govern Wetland Methanogenesis Temperature Response

**DOI:** 10.1002/advs.202518281

**Published:** 2026-08-12

**Authors:** Shuying Qiu, Qingfang Jing, Xuhui Zhou, Xiaoxin Sun, Zhenyu Wei, Baizhi Jiang, Hongyang Chen

**Affiliations:** ^1^ Institute of Carbon Neutrality School of Ecology Key Laboratory of Sustainable Forest Ecosystem Management‐Ministry of Education Northeast Forestry University Harbin China; ^2^ Heilongjiang Sanjiang Plain Wetland Ecosystem Observation and Research Station School of Forestry Northeast Forestry University Harbin China

**Keywords:** carbon substrate, inorganic terminal electron acceptors, temperature sensitivity, wetland CH_4_‐climate feedbacks, wetland microbial methanogenesis

## Abstract

Wetlands constitute Earth's largest natural methane (CH_4_) source, yet projections of their climate feedback remain uncertain due to poorly constrained temperature sensitivity (Q_10‐CH4_) of microbial methanogenesis. While water table dynamics have been demonstrated to regulate wetland methanogenesis by altering key soil conditions such as inorganic terminal electron acceptors (TEAs) and carbon substrates, the influence of these biogeochemical factors on Q_10‐CH4_ remains unclear, particularly at continental scales. Here, we assess Q_10‐CH4_ across 69 wetlands (276 soil samples) spanning diverse climatic and hydrological regimes. We find that Q_10‐CH4_ varies substantially (1.12‐7.75) independent of climatic factors, instead being hierarchically controlled by soil TEAs and carbon‐to‐nitrogen (C/N) ratio. Soil TEAs serve as the overarching determinant that dampens Q_10‐CH4_ at a large scale through thermodynamic constraints. Notably, the promoting effect of C substrate quality (C/N ratio) on temperature sensitivity is conditional, emerging as a dominant driver only when soil TEA concentrations are sufficiently low to alleviate thermodynamic suppression. Our findings suggest that the intrinsic biogeochemical state of wetland soils dictates the magnitude of microbial‐driven CH_4_ emission responses to warming, with critical implications for predicting wetland CH_4_‐climate feedbacks.

## Graphical abstract

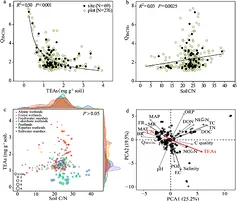

## 1 Introduction

Methane (CH_4_) is a potent greenhouse gas that has contributed to ∼25% of Earth's warming since pre‐industrial times [1, 2, 3]. Wetlands are the largest natural biogenic source of CH_4_, primarily as a result of microbial methanogenesis [4, 5, 6]. Rising temperatures due to climate warming are expected to promote CH_4_ emissions from wetland soils, which would trigger a positive climate change—C cycle feedback [7, 8]. However, the strength of this feedback remains uncertain, primarily due to the elusive response of microbial methanogenesis to changing temperatures in the face of complex biogeochemical properties of wetlands [9, 10].

Methanogenesis is governed by anaerobic soil conditions and the availability of carbon (C) substrates [11]. Environments with higher anaerobic conditions are generally characterized by lower levels of inorganic terminal electron acceptors (TEAs), which are essential for CH_4_ production to occur [4, 12]. Based on thermodynamic theory, CH_4_ production will be competitively suppressed by more favorable TEA‐reducing processes until those TEAs have been consumed [12, 13, 14]. Moreover, methanogenesis is hierarchically regulated by both TEAs and C substrates in wetlands [14]. That is, C substrates only play an important role when soil TEAs are very low [4]. Given that ambient temperature directly affects both TEAs reduction and C substrate availability [15], it is reasonable to expect that these factors may influence the temperature response of microbial methanogenesis. Wetland soils are highly variable in TEAs and C substrates at a contential scale [14]. Until now, however, there has been no large‐scale evaluation of the role of TEAs and C substrates in controlling the temperature response of microbial CH_4_ production, adding considerable uncertainty into predictions of wetland CH_4_ emissions in a warming world [16].

In this study, a survey was established to examine the temperature response of microbial methanogenesis across climatic conditions and variation in soil TEAs and C substrates from diverse wetland ecosystems. Specifically, we collected 276 soil samples from 69 wetland sites, encompassing a broad gradient of climate zones, soil properties, and methanogen communities (Table S1). We evaluated the temperature sensivity of microbial methanogenesis, referred to as Q_10‐CH4_ (a relative increase in soil CH_4_ production rate for a 10°C increase in temperature), by assaying the rates at three temperatures (5°C, 15°C and 25°C) that the wetland soils mostly experienced. Moreover, we examined the associations of Q_10‐CH4_ with various biotic and abiotic variables to identify the key drivers of wetland methanogenesis temperature response at a continental scale.

## 2 Results and Discussion

Across all sampling sites, the Q_10‐CH4_ of soil microbial methanogenesis was highly variable (Figure 1a,b), with values ranging from 1.12 to 7.75 (Figure 1c). However, while the response of microbial respiration to warming in terrestrial soils tends to show a strong climate‐related spatial pattern [17, 18, 19, 20], we found no relationship between Q_10‐CH4_ values and climate variables (Figure S1). Among different wetland types, substantial difference in Q_10‐CH4_ was observed (Figure S2), indicating that the intrinsic characteristics of wetland ecosystems, such as soil physico‐chemical properties and microbial communities, likely underlie the temperature response of microbial methanogenesis at a large scale [14, 16].

**FIGURE 1:**
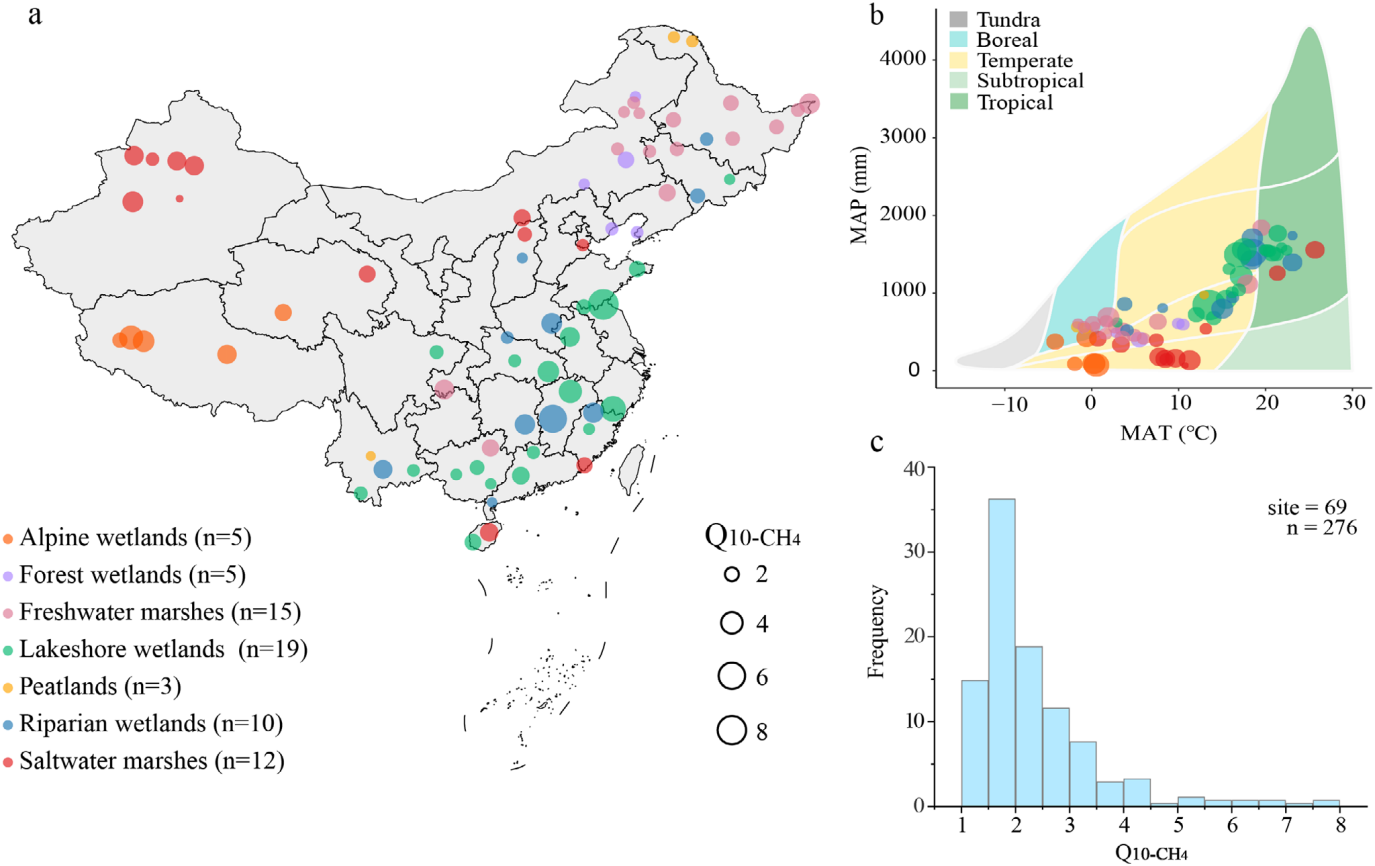
The biogeographic variation of temperature sensitivity of microbial methanogenesis (Q_10‐CH4_). (a) Points indicating the temperature sensitivity of the CH_4_ production rates from 69 wetland sites across China. (b) Distribution of sampling sites across variation in mean annual temperatures (MAT) and mean annual precipitation (MAP), superimposed over Whittaker biomes [21]. In both (a) and (b), point colors represent wetland types classified according to the Ramsar Convention (State Forestry Administration, 2015), and point size is proportional to the value of the measured Q_10‐CH4_, with larger size representing higher value. (c) Frequency distribution of the temperature sensitivity of wetland soil methanogenesis.

Among all variables, soil environments and C substrate together explained 74% of the variance in Q_10‐CH4_ (Figure 2a). The remaining variation was explained by variation in climate and microbial community (Figure 2a). By disentangling the independent effects of all factors, we found that spatial variation in Q_10‐CH4_ was predominantly determined by inorganic terminal electron acceptors (TEAs, which are the sum of sulfate, nitrate, and iron(III)) and the soil C/N ratio (Figure 2b). These two factors independently accounted for 46.7% and 13.6% of the observed spatial variation in Q_10‐CH4_, respectively (Figure S3). These findings highlight the importance of considering both TEAs availability and C substrates when predicting the response of large‐scale wetland CH_4_ emissions to warming.

**FIGURE 2:**
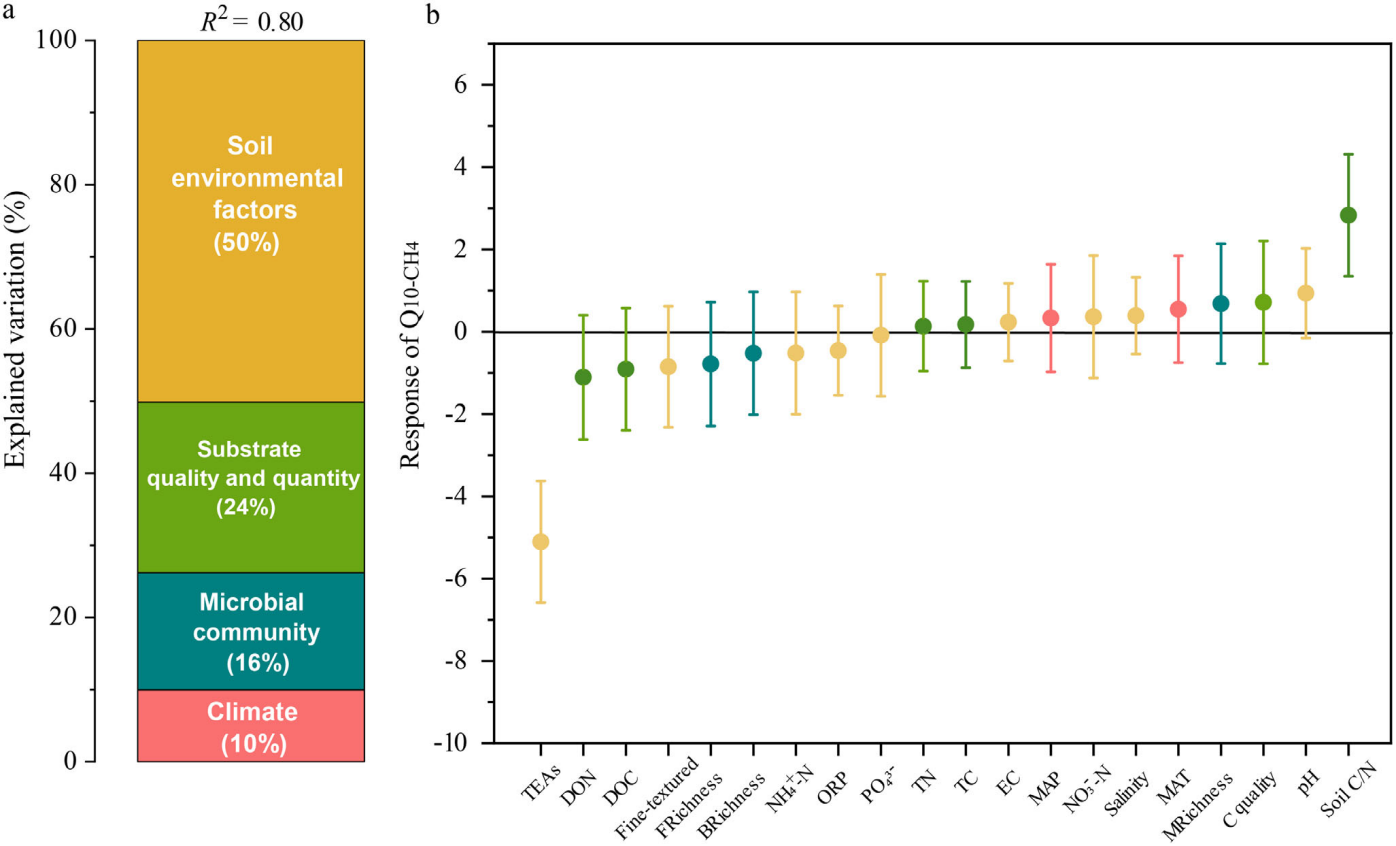
Predictors of temperature sensitivity of microbial methanogenesis (Q_10‐CH4_). (a) Results from the random forest analysis (n = 276), which shows the relative importance of the different drivers in predicting Q_10‐CH4_. (b) The response of Q_10‐CH4_ to influencing variables estimated by ridge regression (n = 276). Circles represent the standardized coefficient of ridge regression between Q_10‐CH4_ and each variable; bars indicate 95% CIs. The influencing variables are: TEAs (soil inorganic terminal electron acceptors, mg/g); DON (dissolved organic nitrogen, mg/g); DOC (dissolved organic carbon, mg/g); Fine‐textured (%); FRichness (fungi richness); BRichness (bacteria richness); NH_4_^+^‐N (ammonium nitrogen, mg/kg); ORP (oxidation‐reduction potential, mV); PO_4_^3−^ (phosphate, mg/g); TN (total nitrogen, mg/g); TC (total carbon, mg/g); EC (electrical conductivity, µS/cm); MAP (mean annual precipitation, mm); NO_3_^−^‐N (nitrate nitrogen; mg/kg); salinity (ppt); MAT (mean annual temperature, °C); MRichness (methanogen richness).

Specifically, we observed a significant negative correlation (*p* < 0.001) between TEAs and Q_10‐CH4_ across all wetland sites (Figure 3a). Further evaluation of the individual effects of the three primary TEAs (i.e., sulfate, nitrate, and iron(III)) on the spatial variation in Q_10‐CH4_ revealed that all three TEAs exhibited consistent and significant negative relationships with Q_10‐CH4_ (Figure S4). Moreover, their respective effects were statistically independent, as supported by the multi‐collinearity test (Table S2). These results demonstrate that soils with inherently lower TEA concentrations have a greater temperature sensitivity of microbial methanogenesis compared to soils with naturally higher TEA concentrations (Figure 3a). This pattern is largely because, in soils with lower TEA availability, the concentrations of both individual and total TEAs decline significantly faster at higher temperature (Figure S5), suggesting that elevated temperatures accelerate microbial reduction of TEAs [22, 23, 24, 25]. This rapid depletion attenuates the inhibitory effect of TEAs on methanogenesis at higher temperatures, leading to a higher Q_10‐__CH4_ [4, 14]. Conversely, in soils with higher TEA concentrations, which also possess lower methanogen taxonomic richness (Figure S6), these TEAs consistently suppressed methanogenic activity through unfavorable soil redox dynamics, regardless of ambient temperature fluctuation [26, 27]. This electron acceptor‐focused perspective offers a more nuanced understanding of the response of methanogenesis to changing temperatures than simple redox potential measurements, which may overlook local heterogeneity in TEAs availability [27].

**FIGURE 3:**
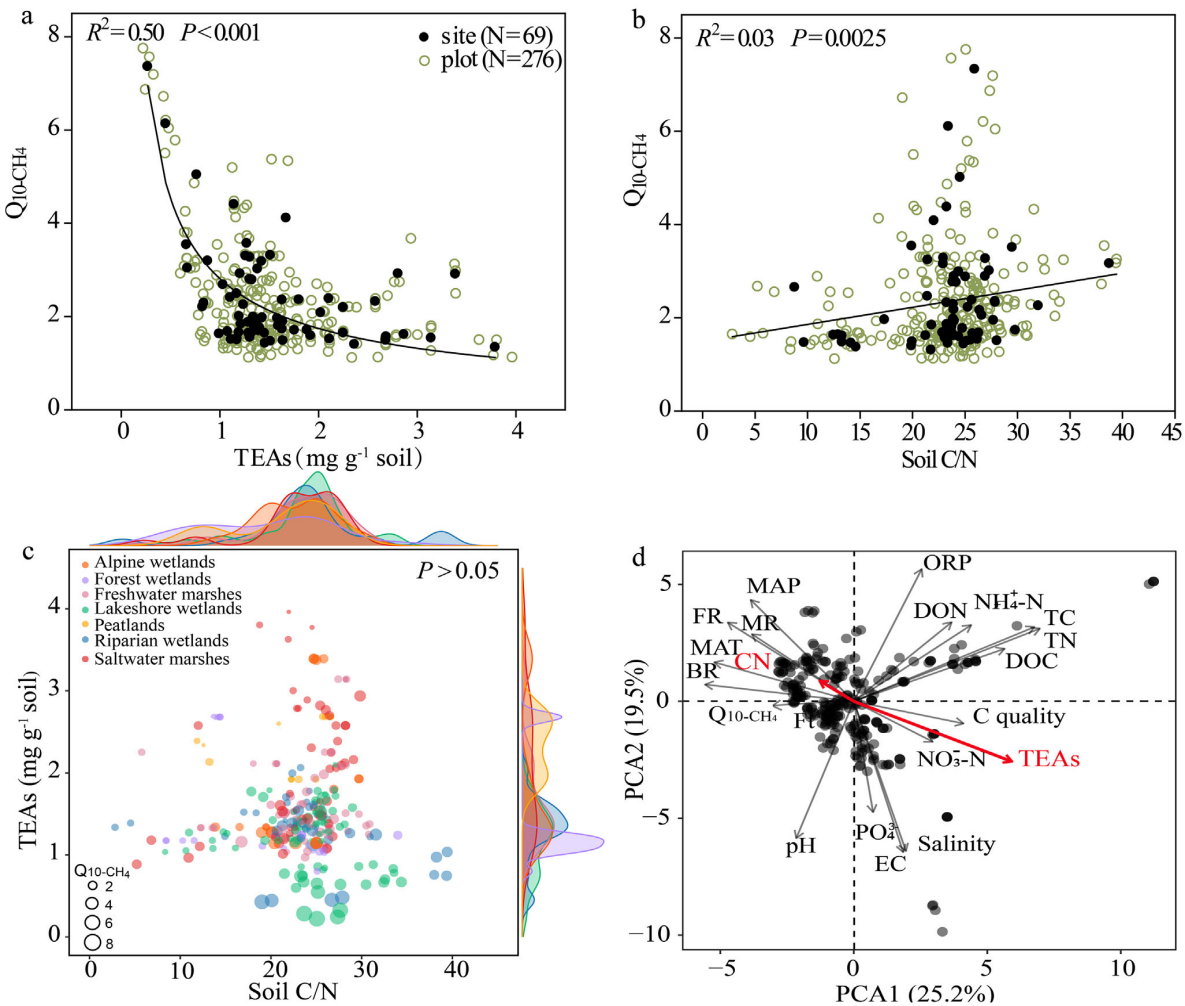
The role of soil inorganic terminal electron acceptors (TEAs) and soil C/N ratio in regulating temperature sensitivity of microbial methanogenesis (Q_10‐CH4_). (a) Relationship between TEAs and Q_10‐CH4_ values across wetland sites (n = 276). The fitted solid line was estimated from a mixed‐effects model. (b) Relationship between soil C/N ratio and Q_10‐CH4_ values across wetland sites (n = 276). The fitted solid line was estimated from a mixed‐effects model.(c) Relationship between TEAs concentration and soil C/N ratio for all sampling sites. Point colors indicate different wetland types, and point sizes are proportional to the corresponding Q_10‐CH4_ values, with larger points representing higher values. Frequency distribution histograms of TEAs and soil C/N ratio are shown for each wetland type, with colors representing the respective wetland categories. (b) Principal component analysis for the Q_10‐CH4_. Ft (Fine‐textured, %); FR (fungi richness); BR (bacteria richness); MR (methanogen richness); CN (soil C/N ratio). The abbreviation refers to Figure 2.

In addition, we found a significant positive correlation between the soil C/N ratio and Q_10‐CH4_ (*p* < 0.01; Figure 3b). This result indicates that the temperature sensitivity of microbial methanogenesis may increase with the complexity of the C substrate in soil organic matter, consistent with the carbon quality‐temperature (CQT) hypothesis [28]. We further analyzed the relationship between the DOC/DON ratio, as a proxy for the quality of labile organic matter, and Q_10‐CH4_. However, no significant correlation was observed (*p* > 0.05; Figure S7). This lack of correlation likely stems from the inherent temporal instability and pronounced seasonal variability of wetland dissolved organic matter pools [29]. Thus, snapshot measurements of DOC and DON may not accurately represent the integrated availability of labile C and N substrates over the long term [29], potentially obscuring their relationship with Q_10‐CH4_ at a large spatial scale.

Notably, there is not a significant interaction effect between TEAs and the soil C/N ratio on Q_10‐CH4_ (*p* > 0.05; Figure 3c), supporting the finding that the TEAs and soil C/N ratio independently modulate the temperature response of microbial methanogenesis from two distinct dimensions (Figure 3d). This lack of interaction is likely due to the decoupling of these two factors in influencing Q_10‐CH4_, which are separately governed by distinct environmental drivers across diverse wetland soils. The concentration and composition of TEAs are closely related to hydrological dynamics and sediment properties, which are highly variable across wetlands [4]. In contrast, the soil C/N ratio is predominantly influenced by vegetation inputs and microbial metabolic processes [30, 31, 32, 33], with similar distributions of soil C/N ratios across diverse wetland types (Figure 3c). The stronger explanatory power of TEA concentrations on Q_10‐CH4_ (*R* ^2^ = 0.50; Figure 3a) suggests that their geographic variability plays a stronger role in regulating the temperature response of microbial methanogenesis, whereas the more uniform distribution of C/N ratios across wetland types contributes less to variation in Q_10‐CH4_ (*R* ^2^ = 0.03; Figure 3b).

Given the hierarchical effects of TEAs and C substrates on microbial methanogenesis [4], we employed partial dependence plots to quantify the sensitivity of Q_10‐CH4_ to the soil C/N ratio across the observed TEAs gradient. Our analysis identified a distinct threshold effect, where Q_10‐CH4_ exhibited a markedly higher sensitivity to C/N variations at TEA concentrations below 1.35 mg g^−1^ soil (*p* < 0.001; Figure 4a). Based on this threshold, we partitioned the wetland sites into low‐TEAs and high‐TEAs groups and used linear mixed‐effects models to evaluate the relationship between Q_10‐CH4_ and soil C/N ratio in each group. The soil C/N ratio exerted a significant positive influence on Q_10‐CH4_ in the low‐TEAs group (*R* ^2^ = 0.12; *p* < 0.05), whereas this relationship became non‐significant in the high‐TEAs group (*p* > 0.05; Figure 4b). Extended analyses further confirmed that Q_10‐CH4_ values were highest when TEAs concentration was low, and soil C/N ratio was high (Figure S8). These results suggest that the thermodynamic constraint imposed by TEAs serves as the primary factor in regulating the spatial variation of Q_10‐CH4_, and the modulating influence of substrate quality (C/N ratio) emerges as a major driver when this primary TEA limitation is sufficiently alleviated.

**FIGURE 4:**
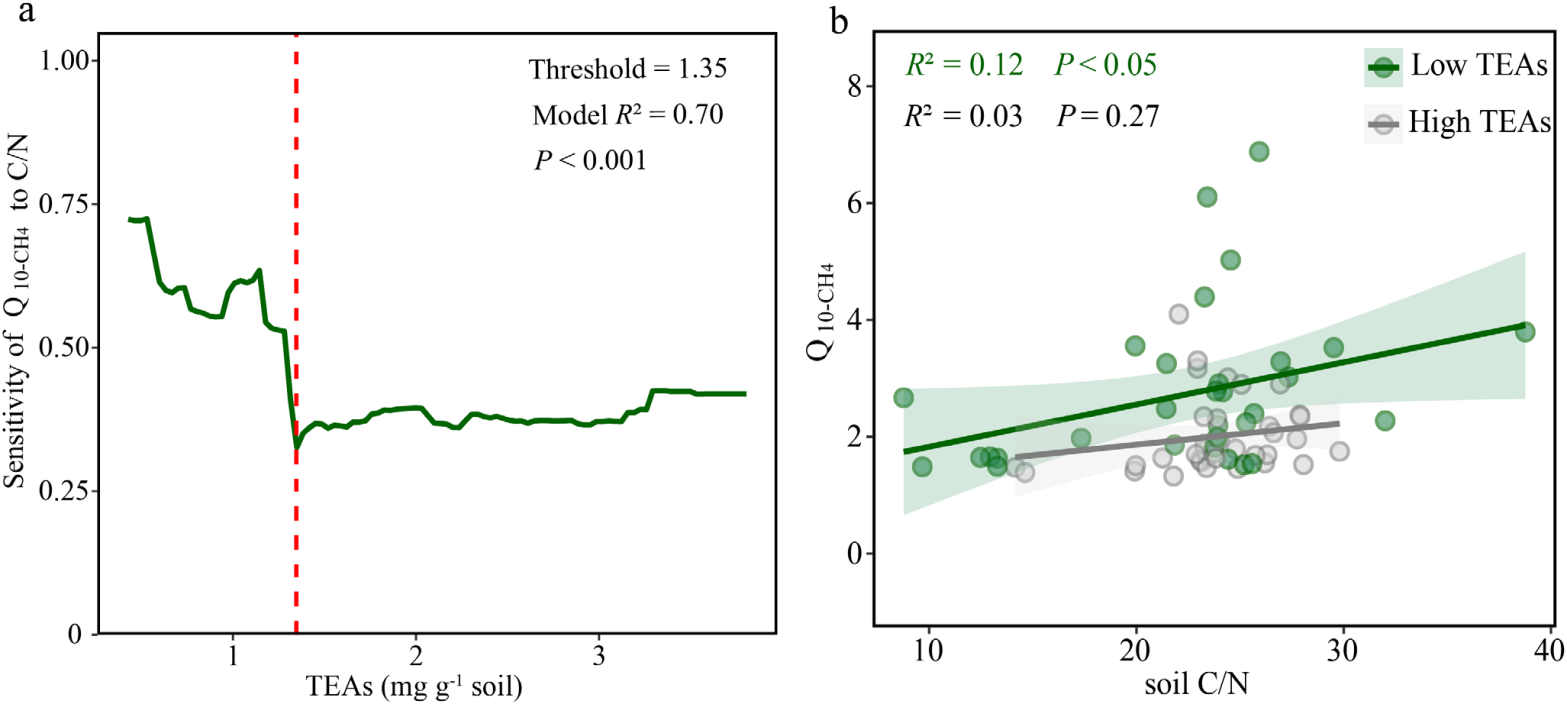
Threshold effect of terminal electron acceptors (TEAs) on the relationship between soil C/N ratio and the temperature sensitivity of microbial methanogenesis (Q_10‐CH4_). (a) Sensitivity analysis based on Partial Dependence Plots (n = 276). The solid dark green line represents the sensitivity of Q_10‐CH4_ to variations in the soil C/N ratio across the TEA concentration gradient. The red dashed line indicates the critical TEA threshold identified at 1.35 mg g^−1^ soil. (b) The relationships between Q_10‐CH4_ and soil C/N ratio under contrasting TEAs levels. 69 wetland sites were categorized into low TEAs (TEAs < 1.35 mg g^−1^ soil, n = 32) and high TEAs (TEAs > 1.35 mg g^−1^ soil, n = 37) groups based on the identified TEA threshold. A linear mixed‐effect model was used to evaluate the relationship between Q_10‐CH4_ and soil C/N ratio in each group, with site as a random effect. Solid lines indicate linear fits, and shaded areas represent the 95% confidence intervals.

Overall, our results provide robust empirical support for the idea that the continental‐scale variability in the temperature response of wetland methanogenesis is hierarchically governed by soil TEAs and C substrate availability. In particular, soil TEAs act as the overarching determinant that dampens the temperature sensitivity of methanogenesis across diverse wetlands, while the promoting effect of C substrate quality on temperature sensitivity is conditional, emerging as a dominant driver only in wetlands with lower soil TEAs. By reconciling microbial‐scale mechanisms with continental‐scale patterns, our findings emphasize the need to incorporate spatial variability in both TEAs and C substrates to improve predictions of wetland CH_4_ emissions under climate warming. Furthermore, future research should investigate how global change may disrupt this TEA‐C substrate hierarchy, further refining projections of wetland contributions to global CH_4_ budgets.

## 3 Experimental Section

### 3.1 Study Site and Soil Sampling

In June 2023, we collected 276 soil samples (69 sites × 4 replicates per site) from across China's major zones of wetland distribution (including cold temperate, temperate, subtropical, tropical and alpine zones) (Table S1). These sites span a wide latitudinal range with substantial climate variability. This leads to variation in soil C/N and terminal electron acceptor (TEAs) concentration (the sum of sulfate, nitrate, and iron(III)) among the regions, enabling us to examine the relationships between soil C/N ratio, TEAs, and Q_10‐CH4_ values.

At each site, we established four 5 × 5 m^2^ sampling plots randomly placed more than 20 m apart from each other. We then collected surface soils (0–10 cm) from five random locations within each plot, which we homogenized into one composite sample per plot. For each composite sample, we stored one part at 4°C for soil incubation and microbial measurement, and air‐dried the other part to determine the physicochemical properties after passing it through a 2‐mm sieve and removing roots and gravel.

### 3.2 Soil Laboratory Incubations

We placed ∼ 25 g dry weight of fresh soils into 125 mL glass containers and adjusted the moisture level to 100% of the water‐holding capacity for anaerobic treatments. We flused the headspace of the incubation microcosms with high‐purity N_2_ gas and then preincubated the soil samples at 20°C for a week to minimize the potential effects of disturbance and encourage microbial activity. We incubated microcosms with all soil samles simultaneously under changing temperatures manipulated by a cryogenic thermostatic bath (precision: ± 0.1 °C) at 5°C, 15°C, and 25°C. After changing to a new temperature, the jars were left to equilibrate for several hours to allow them to reach a stable state, thereby avoiding potential effects of temperature fluctuations on microbial CH_4_ production. After the equilibration period, the incubation jars were sealed, 5 mL headspace samples were collected using syringes [17]. We measured the CH_4_ concentrations in the gas samples using a gas chromatograph equipped with a FID detector (Agilent 7890 A, Agilent Technologies Inc., Santa Clara, California, USA) and calculated the CH_4_ emission rate at each temperature as the change in headspace gas concentration, headspace volume, and the dry weight of the soil sample. We fit the mean CH_4_ emission rate at each temperatures to an exponential model:

(1) $$R\;=\;ae^{\mathrm{bT}}$$

where R is the CH_4_ emission rate at temperature T (°C), and a and b are fitted parameters. We then calculated Q_10_ values (the factor by which soil respiration changes with every 10°C increase) as follows:

(2) $$Q_{10}=e^{10b}$$

Additionally, a 40‐day incubation experiment was conducted using marsh wetland soils to evaluate the reduction rates of nitrate (NO_3_^−^), Fe(III), and sulfate (SO_4_^2−^) across a temperature gradient (5°C, 15°C, and 25°C). The concentrations of these TEAs were measured on days 1, 3, 7, 15, 25, and 40 to monitor their respective depletion dynamics.

### 3.3 Soil Property Analyses

We measured soil pH, soil oxidation–reduction potential, and soil electrical conductivity of air‐dried soils in a 1:2.5 (v/v) soil/water ratio using a meter (Conductivity Meter P912); soil TC using an elemental analyzer (Vario TOC cube, Elementar); and soil DOC, DON, and TN concentration using a TOC analyzer (Multi N/C 3100). We measured soil texture after removing organic matter and carbonates [34] using a particle size analyzer (Laser Particle Sizer, LS‐CWM(2), OMEC).

### 3.4 Inorganic Terminal Electron Acceptors

We analyzed NO_3_^−^ and SO_4_^2−^ using an ion chromatograph (ICS‐1100, Dionex, USA). Soil Fe(II) and Fe(III) concentrations using the ferrozine‐ultraviolet absorbance method [35]. Specifically, we extracted ∼0.2 g of fresh bulk soil with 5 ml of 0.5‐M HCl overnight and measured Fe(II) by absorbance at 562 nm on a UV‐Vis spectrometer (Shimadzu UV‐2550) after mixing with 5 mm ferrozine solution. We first resduced total Fe using hydroxylamine hydrochloride (2%) and then measured it as Fe(II) as above. We calculated Fe(III) by subtracting Fe(II) from total Fe and expressing it as total mg g^−1^ soil.

### 3.5 Substrate Quality

We determined soil C quality using Fourier transform infrared spectroscopy (FTIR, Nicolet iS5, Thermo Scientific). To do so, we sieved air‐dried soil to 0.15 mm and further homogenised it by grinding with an agate mortar and pestle. We obtained reflectance spectra (400–4000 cm^−1^) using FTIR, and relative peak areas were calculated as the areas of distinct reflectance peaks. Relative peak areas reflect the relative abundance of different organic C functional groups, such as aromatic (∼1635 cm^−1^) and poly‐saccharides (∼1000 cm^−1^) C groups. We calculated the ratio of aliphatics to poly‐saccharides (rA1635:rA1000 ratio) to represent soil C quality (a high ratio was considered to represent a poor C quality) [36].

### 3.6 Microbial Properties

To assess the richness of bacterial, fungal, and methanogen communities, amplicon sequencing was conducted using the Illumina MiSeq platform (Illumina Inc., San Diego, CA, USA). Soil DNA was extracted using the Powersoil DNA Isolation Kit (Mo Bio, USA) following the manufacturer's instructions. The richness, defined as the number of amplicon sequence variants (ASVs), was calculated from rarefied ASV abundance tables for bacteria, fungi, and methanogens. Bioinformatic processing was performed using a combination of QIIME 2 v2022.2.0 pipeline. DNA extraction was carried out using the ABI GeneAmp PCR System 9700 (Life Technologies, Thermo Fisher Scientific, USA). The V3–V4 region of the bacterial 16S rRNA gene was amplified with the forward primer 338F (5'ACTCCTACGGGAGGCAGCAG3') and the reverse primer 806R (5'GGACTACHVGGGTWTCTAAT3') [37], and the fungal ITS1 region was amplified using primer ITS1F (5'CTTGGTCATTTAGAGGAAGTAA3') and the reverse primer ITS2R (5'CTTGGTCATTTAGAGGAAGTAA3') [38]. For mcrA amplification, primer pairs (the forward primer is 5'GGTGGTGTMGGATTCACACARTAYGCWACAGC3', the revise primer is 5'TTCATTGCRTAGTTWGGRTAGTT3') [39].

### 3.7 Data Analysis

All statistical analyses were performed using the software R 4.4.0. We identified the main predictors of Q_10‐CH4_ using random forest analysis [40] among the following variables: MAT, MAP, soil pH, EC, salinity, ORP, fine‐textured, soil TEAs, NH_4_^+^, NO_3_^−^, soil TC, TN, soil C/N ratio, DOC, DON, C quality, and soil microbial community composition (the richness of bacteria, fungi, and methanogens). To account for multicollinearity among those factors, we performed a ridge regression analysis to verify the effect of all factors on Q_10‐CH4_ [41]. The relative contribution of each variable was determined using relative weight analysis [42]. Pearson correlation analysis was conducted to identify the positive or negative correlations among the explanatory factors as well as between each factor and Q_10‐CH4_. To evaluate the relationships between Q_10‐CH4_ and each variable, a mixed‐effects model with the sampling site as a random factor was performed with “nlme” package [43]. One‐way ANOVA was conducted to examine differences in Q_10‐CH4_ across different wetland types. Additionally, partial dependence plots were employed to identify the threshold‐dependent regulatory effects of TEAs on the relationship between soil C/N ratio and Q_10‐CH4_ [44].

## Author Contributions

H.C. developed the original ideas presented in the manuscript; S.Q. and Q.J. conducted the measurements with the assistance from X.S. and other lab assitants; S.Q. analyzed the data with the assistance from H.C. and X.Z.; S.Q. and H.C. wrote the first draft, and all authors jointly revised the manuscript.

## Conflicts of Interest

The authors declare no conflicts of interest.

## Supporting information




**Supporting File**: advs76728‐sup‐0001‐SuppMat.docx.

## Data Availability

All data that support the findings of this study can be found in the article and/or Supporting Information.

## References

[1] S. Kirschke , P. Bousquet , P. Ciais , M. Saunois , and G. Zeng , “Three Decades of Global Methane Sources and Sinks,” Nature Geoscience 6, no. 10 (2013): 813–823, 10.1038/ngeo1955.

[2] G. Yvon‐Durocher , A. Allen , D. Bastviken , et al., “Methane Fluxes Show Consistent Temperature Dependence Across Microbial to Ecosystem Scales,” Nature 507, no. 7493 (2014): 488–491, 10.1038/nature13164. 24670769

[3] Z. Zhang , B. Poulter , A. F. Feldman , et al., “Recent Intensification of Wetland Methane Feedback,” Nature Climate Change 13, no. 5 (2023): 430–433, 10.1038/s41558-023-01629-0.

[4] S. D. Bridgham , H. Cadillo‐Quiroz , J. K. Keller , and Q. Zhuang , “Methane Emissions From Wetlands: Biogeochemical, Microbial, and Modeling Perspectives From Local to Global Scales,” Global Change Biology 19, no. 5 (2013): 1325–1346, 10.1111/gcb.12131. 23505021

[5] C. Knoblauch , C. Beer , S. Liebner , M. N. Grigoriev , and E. M. Pfeiffer , “Methane Production as Key to the Greenhouse Gas Budget of Thawing Permafrost,” Nature Climate Change 8, no. 4 (2018): 309–312, 10.1038/s41558-018-0095-z.

[6] R. Segers , “Methane Production and Methane Consumption: A Review of Processes Underlying Wetland Methane Fluxes,” Biogeochemistry 41, no. 1 (1998): 23–51, 10.1023/A:1005929032764.

[7] W. J. Mitsch , B. Bernal , A. M. Nahlik , et al., “Wetlands, Carbon, and Climate Change,” Landscape Ecology 28, no. 4 (2013): 583–597, 10.1007/s10980-012-9758-8.

[8] M. Saunois , A. R. Stavert , B. Poulter , et al., Earth Syst Sci Data 12 (2024): 1561–1623, 10.5194/essd-12-1561-2020.

[9] A. A. Bloom , P. I. Palmer , A. Fraser , D. S. Reay , and C. Frankenberg , “Large‐scale Controls of Methanogenesis Inferred From Methane and Gravity Spaceborne Data,” Science 327, no. 5963 (2010): 322–325, 10.1126/science.1175176. 20075250

[10] T. Bao , G. Jia , and X. Xu , “Wetland Heterogeneity Determines Methane Emissions: A Pan‐Arctic Synthesis,” Environmental Science & Technology 55, no. 14 (2021): 10152–10163, 10.1021/acs.est.1c01616. 34229435

[11] T. R. Christensen , A. Ekberg , L. Ström , et al., “Factors Controlling Large Scale Variations in Methane Emissions From Wetlands,” Geophysical Research Letters 30, no. 7 (2003): 1414, 10.1029/2002GL016848.

[12] Z. Wang , R. D. Delaune , W. H. Patrick , and P. H. Masscheleyn , “Soil Redox and pH Effects on Methane Production in a Flooded Rice Soil,” Soil Science Society of America Journal 57, no. 2 (1993): 382–385, 10.2136/sssaj1993.03615995005700020016x.

[13] E. M. D'Angelo and K. R. Reddy , “Regulators of Heterotrophic Microbial Potentials in Wetland Soils,” Soil Biology and Biochemistry 31, no. 6 (1999): 815–830, 10.1016/S0038-0717(98)00181-3.

[14] S. Cui , P. Liu , H. Guo , et al., “Wetland Hydrological Dynamics and Methane Emissions,” Communications Earth & Environment 5, no. 1 (2024): 470, 10.1038/s43247-024-01635-w.

[15] K. S. Inglett , P. W. Inglett , K. R. Reddy , and T. Z. Osborne , “Temperature Sensitivity of Greenhouse Gas Production in Wetland Soils of Different Vegetation,” Biogeochemistry 108, no. 1‐3 (2012): 77–90, 10.1007/s10533-011-9573-3.

[16] S. A. Comer‐Warner , P. Romeijn , D. C. Gooddy , et al., “Thermal Sensitivity of CO_2_ and CH_4_ Emissions Varies With Streambed Sediment Properties,” Nature Communications 9, no. 1 (2018): 2803, 10.1038/s41467-018-04756-x. PMC6052154 30022025

[17] J. Li , M. Nie , E. Pendall , et al., “Biogeographic Variation in Temperature Sensitivity of Decomposition in Forest Soils,” Global Change Biology 26, no. 3 (2019): 1873–1885, 10.1111/gcb.14838. 31518470

[18] Q. Wang , S. Liu , and P. Tian , “Carbon Quality and Soil Microbial Property Control the Latitudinal Pattern in Temperature Sensitivity of Soil Microbial Respiration Across Chinese Forest Ecosystems,” Global Change Biology 24, no. 7 (2018): 2841–2849, 10.1111/gcb.14105. 29476638

[19] N. Fierer , B. P. Colman , J. P. Schimel , and R. B. Jackson , “Predicting the temperature dependence of microbial respiration in soil: A continental‐scale analysis,” Global Biogeochemical Cycles 20, no. 3 (2006): GB3026, 10.1029/2005GB002644.

[20] J. Feng , J. Wang , Y. Song , and B. Zhu , “Patterns of Soil Respiration and Its Temperature Sensitivity in Grassland Ecosystems Across China,” Biogeosciences 15, no. 17 (2018): 5329–5341, 10.5194/bg-15-5329-2018.

[21] R. H. Whittaker , Communities and Ecosystems (Macmillan, 1970).

[22] A. E. Sutton‐Grier , J. K. Keller , R. Koch , C. Gilmour , and J. P. Megonigal , “Electron Donors and Acceptors Influence anaerobic Soil Organic Matter Mineralization in Tidal Marshes,” Soil Biology and Biochemistry 43, no. 7 (2011): 1576–1583, 10.1016/j.soilbio.2011.04.008.

[23] J. E. Sawicka , B. B. Jørgensen , and V. Brüchert , “Temperature Characteristics of Bacterial Sulfate Reduction in Continental Shelf and Slope Sediments,” Biogeosciences 9, no. 8 (2012): 3425–3435, 10.5194/bg-9-3425-2012.

[24] J. T. Westrich and R. A. Berner , “The Effect of Temperature on Rates of Sulfate Reduction in Marine Sediments,” Geomicrobiology Journal 6, no. 2 (1988): 99–117, 10.1080/01490458809377828.

[25] K. Schilling , T. Borch , C. C. Rhoades , and C. Pallud , “Temperature Sensitivity of Microbial Fe(III) Reduction Kinetics in Subalpine Wetland Soils,” Biogeochemistry 142, no. 1 (2019): 19–35, 10.1007/s10533-018-0520-4.

[26] C. Soued , M. J. Bogard , K. Finlay , et al., “Salinity Causes Widespread Restriction of Methane Emissions From Small Inland Waters,” Nature Communications 15, no. 1 (2024): 717, 10.1038/s41467-024-44715-3. PMC10808391 38267478

[27] H. Chen , X. Xu , C. Fang , B. Li , and M. Nie , “Differences in the Temperature Dependence of Wetland CO_2_ and CH_4_ Emissions Vary With Water Table Depth,” Nature Climate Change 11, no. 9 (2021): 766–771, 10.1038/s41558-021-01108-4.

[28] E. A. Davidson and I. A. Janssens , “Temperature Sensitivity of Soil Carbon Decomposition and Feedbacks to Climate Change,” Nature 440, no. 7081 (2006): 165–173, 10.1038/nature04514. 16525463

[29] J. Neff and G. Asner , “Dissolved Organic Carbon in Terrestrial Ecosystems: Synthesis and a Model,” Ecosystems 4, no. 1 (2001): 29–48, 10.1007/s100210000058.

[30] M. Schmidt , M. Torn , S. Abiven , et al., “Persistence of Soil Organic Matter as an Ecosystem Property,” Nature 478, no. 7367 (2011): 49–56, 10.1038/nature10386. 21979045

[31] D. Sihi , P. W. Inglett , and K. S. Inglett , “Carbon Quality and Nutrient Status Drive the Temperature Sensitivity of Organic Matter Decomposition in Subtropical Peat Soils,” Biogeochemistry 131, no. 1‐2 (2016): 103–119, 10.1007/s10533-016-0267-8.

[32] H. Hu , J. Chen , F. Zhou , et al., “Relative Increases in CH_4_ and CO_2_ Emissions From Wetlands Under Global Warming Dependent on Soil Carbon Substrates,” Nature Geoscience 17, no. 1 (2024): 26–31, 10.1038/s41561-023-01345-6.

[33] L. Meng , P. G. M. Hess , N. M. Mahowald , et al., “Sensitivity of Wetland Methane Emissions to Model Assumptions: Application and Model Testing Against Site Observations,” Biogeosciences 9, no. 7 (2012): 2793–2819, 10.5194/bg-9-2793-2012.

[34] C. Mao , D. Kou , L. Chen , et al., “Permafrost Nitrogen Status and Its Determinants on the Tibetan Plateau,” Global Change Biology 26, no. 9 (2020): 5290–5302, 10.1111/gcb.15205. 32506764

[35] L. L. Stookey , “Ferrozine—a new spectrophotometric reagent for iron,” Analytical Chemistry 42, no. 7 (1970): 779–781, 10.1021/ac60289a016.

[36] S. B. Hodgkins , M. M. Tfaily , C. K. McCalley , et al., “Changes in Peat Chemistry Associated With Permafrost Thaw Increase Greenhouse Gas Production,” Proceedings of the National Academy of Sciences 111, no. 16 (2014): 5819–5824, 10.1073/pnas.1314641111. PMC4000816 24711402

[37] N. Xu , G. Tan , H. Wang , and X. Gai , “Effect of Biochar Additions to Soil on Nitrogen Leaching, Microbial Biomass and Bacterial Community Structure,” European Journal of Soil Biology 74 (2016): 1–8, 10.1016/j.ejsobi.2016.02.004.

[38] R. I. Adams , M. Miletto , J. W. Taylor , and T. D. Bruns , “Dispersal in Microbes: Fungi in Indoor Air Are Dominated by Outdoor Air and Show Dispersal Limitation at Short Distances,” The ISME Journal 7, no. 7 (2013): 1262–1273, 10.1038/ismej.2013.28. PMC3695294 23426013

[39] C. Zhu , J. Zhang , Y. Tang , Z. Xu , and R. Song , “Diversity of Methanogenic Archaea in a Biogas Reactor Fed With Swine Feces as the Mono‐substrate by mcrA Analysis,” Microbiological Research 166, no. 1 (2011): 27–35, 10.1016/j.micres.2010.01.004. 20116227

[40] L. Breiman , “Random Forests,” Machine learning 45, no. 1 (2001): 5–32, 10.1023/A:1010933404324.

[41] W. Chen , S. Wang , J. Wang , et al., “Evidence for Widespread Thermal Optimality of Ecosystem Respiration,” Nature Ecology & Evolution 7, no. 9 (2023): 1379–1387, 10.1038/s41559-023-02121-w. 37488227

[42] J. Peng , S. Xie , J. Tang , et al., “Global Evidence of the Unimodal Response of Ecosystem Respiration to Soil Moisture,” Advanced Science 13, no. 3 (2026): 09753, 10.1002/advs.202509753. PMC12806489 41168960

[43] J. Pinheiro and D. Bates , Mixed‐Effects Models in S and S‐PLUS (Springer Science & Business Media, 2006).

[44] T. Hastie , R. Tibshirani , and J. Friedman , The Elements of Statistical Learning, 2nd Ed (Springer, 2009).

