# Supplementary material for: Soil Inorganic Electron Acceptors and Carbon Substrates Hierarchically Govern Wetland Methanogenesis Temperature Response
Source: Adv Sci (Weinh). 2026 Aug 12;13(57):e18281. doi: 10.1002/advs.202518281 (PMC13464452; doi:10.1002/advs.202518281)
Supplement: Supplementary file 1 [file ADVS-13-e18281-s001.docx]

**Supporting Information**

**Soil Inorganic Electron Acceptors and Carbon Substrates Hierarchically Govern Wetland Methanogenesis Temperature Response**

**Qiu et al. 2025**

**Supplementary Figures**

**Figure S1. The relationship between temperature sensitivity of microbial methanogenesis (Q_10-CH₄_) and climate factors.** Point colors indicate different wetland types. Mean annual temperatures (MAT, °C); mean annual precipitations (MAP, mm).

**Figure S2. The differences in the temperature sensitivity of microbial methanogenesis (Q_10-CH₄_) across diverse wetland types.**

**Figure S3.** **Relative importance of key drivers for the temperature sensitivity of microbial methanogenesis**. The relative contribution of each variable was determined using relative weight analysis. TEAs (soil inorganic terminal electron acceptors, mg/g); DON (dissolved organic nitrogen, mg/g); DOC (dissolved organic carbon, mg/g); Fine-textured (%); FRichness (fungi richness); BRichness (bacteria richness); NH_4_^+^-N (ammonium nitrogen, mg/kg); ORP (oxidation-reduction potential, mV); PO_4_^3-^ (phosphate, mg/g); TN (total nitrogen, mg/g); TC (total carbon, mg/g); EC (electrical conductivity, μS/cm); MAP (mean annual precipitation, mm); NO_3_^-^-N (nitrate nitrogen; mg/kg); salinity (ppt); MAT (mean annual temperature, °C); MRichness (methanogen richness).

**Figure S4.** The relationship between terminal electron acceptors and the temperature sensitivity of microbial methanogenesis.

**Figure S5.** Reduction dynamics of terminal electron acceptors (TEAs) over time at different temperatures. Values represent the mean reduction rates of nitrate, sulfate and Fe(III) across the temperature gradient. Error bars indicate the standard error of the mean (n=3).

**Figure S6. The relationships between terminal electron acceptors (TEAs) and α-diversity of microbial methanogens**. The α-diversity is indicated by the Shannon index (A), Simpson index (B) and richness of methanogens (C).

**Figure S7.** Relationship between the DOC:DON ratio and the temperature sensitivity of microbial methanogenesis.

**Figure S8. The relationships between soil inorganic terminal electron acceptors (TEAs), soil C/N ratio and the temperature sensitivity of microbial methanogenesis (Q_10-CH₄_) across wetland sites.** Observed values (black points) are Q_10-CH₄_ values, and modeled values (colored surfaces) are predictions from the models fitted with observations.

**Supplementary Tables**

**Table S1. The geographic and climatic information of sampling sites.**

| **No.** | **Climate zone** | **Latitude (°N)** | **Longitude (°E)** | **MAT (℃)** | **MAP (mm)** | **pH** | **Total carbon (g/kg)** | **Total nitrogen (g/kg)** | **soil C/N ratio** | **TEAs (mg/g)** | **Methanogen Richness** |
| --- | --- | --- | --- | --- | --- | --- | --- | --- | --- | --- | --- |
| 1 | Subtropical zone | 18.74 | 108.86 | 20.77 | 150.90 | 4.62 | 37.67 | 1.18 | 32 | 0.84 | 1064 |
| 2 | Subtropical zone | 26.68 | 112.85 | 18.38 | 142.60 | 7.31 | 23.23 | 0.60 | 39 | 0.88 | 805 |
| 3 | Subtropical zone | 30.24 | 114.64 | 17.21 | 121.70 | 7.52 | 8.24 | 0.28 | 30 | 0.66 | 441 |
| 4 | Temperate zone | 32.57 | 116.29 | 15.47 | 91.80 | 7.48 | 6.19 | 0.22 | 27 | 0.67 | 393 |
| 5 | Temperate zone | 40.59 | 112.63 | 3.36 | 33.70 | 8.32 | 37.81 | 1.36 | 28 | 2.11 | 328 |
| 6 | Subtropical zone | 27.06 | 114.99 | 18.47 | 152.20 | 7.35 | 6.67 | 0.29 | 23 | 0.45 | 569 |
| 7 | Temperate zone | 37.14 | 121.45 | 12.03 | 72.30 | 7.55 | 14.55 | 0.61 | 24 | 0.83 | 446 |
| 8 | Subtropical zone | 34.76 | 118.86 | 13.49 | 84.60 | 7.66 | 6.42 | 0.25 | 26 | 0.27 | 754 |
| 9 | Subtropical zone | 23.93 | 117.41 | 21.36 | 125.70 | 7.61 | 16.26 | 0.62 | 26 | 2.25 | 697 |
| 10 | Subtropical zone | 27.72 | 119.61 | 16.80 | 149.50 | 7.29 | 31.47 | 1.28 | 25 | 0.77 | 536 |
| 11 | Temperate zone | 34.60 | 117.36 | 14.04 | 67.90 | 7.66 | 39.33 | 1.42 | 28 | 1.43 | 497 |
| 12 | Temperate zone | 37.88 | 112.65 | 8.17 | 80.60 | 8.12 | 24.12 | 0.97 | 25 | 1.51 | 184 |
| 13 | Temperate zone | 38.75 | 117.28 | 13.13 | 54.00 | 8.23 | 19.57 | 0.85 | 23 | 2.87 | 132 |
| 14 | Subtropical zone | 32.53 | 111.49 | 16.23 | 94.40 | 7.84 | 1.36 | 0.19 | 13 | 1.29 | 304 |
| 15 | Subtropical zone | 39.85 | 119.51 | 10.50 | 59.50 | 7.75 | 26.19 | 1.09 | 23 | 1.08 | 243 |
| 16 | Subtropical zone | 21.43 | 110.33 | 23.11 | 174.00 | 6.62 | 46.03 | 2.15 | 20 | 1.46 | 709 |
| 17 | Temperate zone | 46.71 | 132.13 | 3.57 | 53.40 | 7.60 | 14.79 | 0.62 | 24 | 1.36 | 428 |
| 18 | Subtropical zone | 25.09 | 110.21 | 19.53 | 184.10 | 6.86 | 42.37 | 1.65 | 26 | 1.10 | 709 |
| 19 | Temperate zone | 41.88 | 86.38 | 10.75 | 6.80 | 8.23 | 25.86 | 1.20 | 22 | 3.79 | 322 |
| 20 | Temperate zone | 44.42 | 86.16 | 8.47 | 15.20 | 8.07 | 42.03 | 1.74 | 24 | 1.33 | 153 |
| 21 | Subtropical zone | 29.02 | 106.67 | 17.94 | 111.60 | 6.48 | 14.69 | 0.60 | 24 | 1.38 | 836 |
| 22 | Alpine zone | 32.35 | 81.80 | -1.95 | 8.80 | 8.02 | 37.86 | 2.22 | 17 | 1.34 | 108 |
| 23 | Alpine zone | 36.81 | 100.76 | 0.71 | 41.40 | 8.30 | 33.18 | 1.37 | 24 | 2.58 | 206 |
| 24 | Alpine zone | 32.28 | 83.62 | 0.25 | 9.30 | 7.85 | 109.45 | 5.49 | 20 | 1.27 | 52 |
| 25 | Alpine zone | 32.53 | 82.66 | 0.54 | 7.40 | 7.27 | 16.85 | 0.72 | 23 | 1.14 | 151 |
| 26 | Alpine zone | 31.39 | 90.01 | -0.59 | 42.50 | 9.27 | 33.19 | 1.32 | 25 | 3.39 | 88 |
| 27 | Alpine zone | 34.21 | 94.32 | -4.21 | 37.50 | 8.47 | 16.55 | 0.59 | 28 | 1.80 | 156 |
| 28 | Cold temperate zone | 52.76 | 124.27 | -1.69 | 56.55 | 5.95 | 168.90 | 6.45 | 26 | 2.69 | 180 |
| 29 | Cold temperate zone | 47.63 | 121.61 | 0.34 | 56.63 | 5.48 | 103.94 | 5.21 | 20 | 2.11 | 127 |
| 30 | Cold temperate zone | 48.75 | 121.32 | -0.90 | 54.17 | 5.79 | 111.53 | 7.88 | 14 | 2.68 | 181 |
| 31 | Cold temperate zone | 47.72 | 120.45 | -1.57 | 60.74 | 5.41 | 450.78 | 16.07 | 28 | 3.14 | 302 |
| 32 | Cold temperate zone | 42.86 | 117.39 | 2.79 | 48.99 | 8.16 | 12.03 | 1.27 | 10 | 1.17 | 387 |
| 33 | Cold temperate zone | 52.48 | 125.68 | -0.75 | 55.02 | 4.97 | 37.17 | 1.61 | 23 | 1.92 | 272 |
| 34 | Cold temperate zone | 48.35 | 121.19 | -0.91 | 56.27 | 5.42 | 169.05 | 6.36 | 21 | 2.25 | 160 |
| 35 | Cold temperate zone | 45.23 | 119.95 | 1.60 | 47.59 | 6.38 | 11.44 | 0.88 | 13 | 1.33 | 373 |
| 36 | Cold temperate zone | 44.50 | 120.59 | 5.48 | 40.35 | 7.88 | 5.57 | 0.22 | 25 | 1.24 | 501 |
| 37 | Subtropical zone | 22.03 | 100.26 | 20.19 | 154.80 | 7.18 | 15.06 | 0.65 | 23 | 1.35 | 765 |
| 38 | Temperate zone | 44.78 | 82.89 | 9.63 | 16.10 | 7.72 | 27.88 | 1.05 | 27 | 2.81 | 220 |
| 39 | Subtropical zone | 23.77 | 109.18 | 21.24 | 150.00 | 7.56 | 57.29 | 2.40 | 24 | 1.39 | 646 |
| 40 | Temperate zone | 45.92 | 128.77 | 2.73 | 56.60 | 6.09 | 21.43 | 0.92 | 23 | 1.64 | 541 |
| 41 | Temperate zone | 43.18 | 128.54 | 2.92 | 62.40 | 5.68 | 42.94 | 1.79 | 23 | 1.64 | 427 |
| 42 | Temperate zone | 31.55 | 106.08 | 16.93 | 104.00 | 7.93 | 16.23 | 0.66 | 25 | 1.59 | 584 |
| 43 | Subtropical zone | 24.79 | 113.50 | 20.45 | 154.20 | 5.74 | 11.49 | 0.44 | 26 | 1.76 | 224 |
| 44 | Temperate zone | 39.61 | 121.45 | 9.97 | 60.90 | 8.05 | 8.61 | 0.35 | 24 | 1.00 | 320 |
| 45 | Temperate zone | 45.07 | 122.40 | 5.97 | 41.30 | 9.22 | 10.02 | 0.40 | 26 | 1.40 | 264 |
| 46 | Temperate zone | 44.54 | 84.30 | 8.57 | 14.70 | 7.38 | 47.09 | 2.00 | 24 | 1.63 | 483 |
| 47 | Temperate zone | 45.22 | 124.49 | 4.89 | 45.50 | 8.00 | 17.54 | 0.74 | 24 | 1.22 | 377 |
| 48 | Temperate zone | 47.86 | 133.77 | 1.67 | 63.50 | 6.62 | 7.12 | 0.24 | 30 | 1.40 | 375 |
| 49 | Temperate zone | 44.11 | 87.50 | 7.81 | 17.90 | 7.51 | 21.69 | 0.89 | 24 | 1.21 | 631 |
| 50 | Temperate zone | 39.47 | 112.83 | 7.44 | 39.30 | 7.81 | 9.85 | 0.45 | 22 | 1.29 | 228 |
| 51 | Temperate zone | 42.07 | 126.09 | 3.79 | 85.90 | 6.20 | 35.61 | 1.49 | 24 | 1.59 | 713 |
| 52 | Temperate zone | 48.32 | 128.64 | 0.12 | 61.00 | 6.42 | 44.16 | 1.64 | 27 | 2.02 | 544 |
| 53 | Temperate zone | 45.88 | 126.78 | 4.07 | 52.10 | 7.29 | 8.50 | 0.37 | 23 | 1.89 | 529 |
| 54 | Subtropical zone | 23.22 | 112.53 | 21.41 | 176.80 | 5.51 | 9.94 | 0.46 | 21 | 1.17 | 773 |
| 55 | Tropical zone | 23.64 | 101.97 | 23.10 | 139.60 | 7.37 | 18.13 | 0.76 | 24 | 1.30 | 744 |
| 56 | Temperate zone | 42.28 | 123.75 | 7.61 | 63.20 | 6.36 | 22.29 | 0.95 | 23 | 1.63 | 360 |
| 57 | Subtropical zone | 27.47 | 118.09 | 18.48 | 170.90 | 6.26 | 14.01 | 0.61 | 23 | 1.43 | 989 |
| 58 | Temperate zone | 33.48 | 114.90 | 15.03 | 79.90 | 8.01 | 8.41 | 0.31 | 27 | 1.26 | 570 |
| 59 | Temperate zone | 48.28 | 134.67 | 1.91 | 69.20 | 6.69 | 11.37 | 0.52 | 21 | 1.31 | 207 |
| 60 | Tropical zone | 19.40 | 110.11 | 25.70 | 155.60 | 7.90 | 1.42 | 0.14 | 9 | 1.03 | 214 |
| 61 | Subtropical zone | 28.90 | 116.33 | 17.56 | 156.30 | 6.05 | 10.73 | 0.52 | 22 | 1.67 | 283 |
| 62 | Temperate zone | 41.67 | 82.79 | 11.28 | 13.50 | 7.51 | 20.60 | 0.89 | 23 | 1.51 | 306 |
| 63 | Subtropical zone | 30.96 | 112.06 | 16.16 | 101.10 | 6.35 | 2.50 | 0.20 | 12 | 1.20 | 188 |
| 64 | Subtropical zone | 22.67 | 110.23 | 21.86 | 158.50 | 7.38 | 2.74 | 0.21 | 13 | 1.12 | 496 |
| 65 | Tropical zone | 23.30 | 107.59 | 22.43 | 155.10 | 8.50 | 15.19 | 0.60 | 25 | 1.18 | 987 |
| 66 | Temperate zone | 47.19 | 124.23 | 3.54 | 42.40 | 8.57 | 26.02 | 1.09 | 24 | 1.20 | 550 |
| 67 | Subtropical zone | 23.58 | 104.30 | 15.80 | 131.00 | 6.27 | 29.28 | 1.24 | 24 | 1.45 | 832 |
| 68 | Subtropical zone | 26.37 | 117.77 | 19.90 | 155.70 | 5.67 | 16.29 | 0.64 | 26 | 1.31 | 329 |
| 69 | Subtropical zone | 24.54 | 101.03 | 12.94 | 97.60 | 5.19 | 124.13 | 8.15 | 15 | 2.37 | 62 |

The influencing variables are MAT (mean annual temperature); MAP (mean annual precipitation); TEAs (soil inorganic terminal electron acceptors).

**Table S2.** Variance inflation factors (VIF) for terminal electron acceptors in the multiple linear regression of the temperature sensitivity of microbial methanogenesis.

|  | SO_4_^2-^ | NO_3_^-^ | Fe(III) |
| --- | --- | --- | --- |
| VIF | 1.06 | 1.17 | 1.11 |
